# Change of urinary fluoride and bone metabolism indicators in the endemic fluorosis areas of southern china after supplying low fluoride public water

**DOI:** 10.1186/1471-2458-13-156

**Published:** 2013-02-20

**Authors:** Shaoxian Chen, Boling Li, Shao Lin, Yixiang Huang, Xinhua Zhao, Min Zhang, Yuan Xia, Xiaoheng Fang, Junyi Wang, Syni-An Hwang, Shouyi Yu

**Affiliations:** 1 Department of Epidemiology, School of Public Health and Tropical Medicine, Southern Medical University, 1813 Guangzhou Dadao North, Guangzhou, Guangdong 510515, People’s Republic of China; 2 School of Public Health, Sun Yat-sen University, Zhongshan 2nd Road, Guangzhou 510080, People’s Republic of China; 3 School of Public Health, Guangdong Pharmaceutical University, Jianghai Dadao, Guangzhou 510500, People’s Republic of China; 4 Department of Epidemiology and Biostatistics, School of Public Health, University at Albany, State University of New York, One University Place, Rensselaer, NY 12144-3445, USA

## Abstract

**Background:**

Few studies have evaluated health impacts, especially biomarker changes, following implementation of a new environmental policy. This study examined changes in water fluoride, urinary fluoride (UF), and bone metabolism indicators in children after supplying low fluoride public water in endemic fluorosis areas of Southern China. We also assessed the relationship between UF and serum osteocalcin (BGP), calcitonin (CT), alkaline phosphatase (ALP), and bone mineral density to identify the most sensitive bone metabolism indicators related to fluoride exposure.

**Methods:**

Four fluorosis-endemic villages (intervention villages) in Guangdong, China were randomly selected to receive low-fluoride water. One non-endemic fluorosis village with similar socio-economic status, living conditions, and health care access, was selected as the control group. 120 children aged 6-12 years old were randomly chosen from local schools in each village for the study. Water and urinary fluoride content as well as serum BGP, CT, ALP and bone mineral density were measured by the standard methods and compared between the children residing in the intervention villages and the control village. Benchmark dose (BMD) and benchmark dose lower limit (BMDL) were calculated for each bone damage indicator.

**Results:**

Our study found that after water source change, fluoride concentrations in drinking water in all intervention villages (A-D) were significantly reduced to 0.11 mg/l, similar to that in the control village (E). Except for Village A where water change has only been taken place for 6 years, urinary fluoride concentrations in children of the intervention villages were lower or comparable to those in the control village after 10 years of supplying new public water. The values of almost all bone indicators in children living in Villages B-D and ALP in Village A were either lower or similar to those in the control village after the intervention. CT and BGP are sensitive bone metabolism indicators related to UF. While assessing the temporal trend of different abnormal bone indicators after the intervention, bone mineral density showed the most stable and the lowest abnormal rates over time.

**Conclusions:**

Our results suggest that supplying low fluoride public water in Southern China is successful as measured by the reduction of fluoride in water and urine, and changes in various bone indicators to normal levels. A comparison of four bone indicators showed CT and BGP to be the most sensitive indicators.

## Background

Fluorine is the 13^th^ most abundant naturally occurring element in the earth’s crust [1]. It is a highly reactive element which combines with other elements and molecules to form fluorides in the Earth’s surface [2]. While scientists are still uncertain whether fluoride is essential to human health, many believe that small amounts of fluoride in the diet can help prevent dental caries and strengthen bones [3]. On the other hand, chronic ingestion of high doses of fluoride has a number of adverse effects on human health, including dental fluorosis and skeletal fluorosis [4,5], increased bone fractures, decreased birth rates, increased rates of urolithiasis (kidney stones), impaired thyroid function, and lower intelligence in children [6,7]. In China, there are three major sources of excessive fluoride: drinking water, coal pollution, and drinking brick tea infusions, which lead to endemic dental and skeletal fluorosis [8-13]. According to China’s national prevention plan (2004-2010) [14] for key endemic diseases, in the year 2003, there were about 3 million people suffering from skeletal fluorosis due to exposure during childhood to excessive fluoride in local underground water. Our study, conducted in 2001-2002 in Guangdong province, showed that there were 396 villages with drinking water- related endemic fluorosis affecting a total population of 502,400. In Shantou City of Guangdong, there were 73 villages (population of 216,000) in the endemic fluorosis areas [15,16]. The water fluoride concentrations were much higher than the national standard of 1.0 mg/l, ranging from 1.0 mg/l to 11.6 mg /l, and the prevalence of dental fluorosis was 62% among children aged 8-15 years [17-19].

As high fluoride drinking water seriously endangers local residents’ health, Shantou was recognized as an endemic fluorosis city in 1980. To address health risks associated with fluoride, public water with lower fluoride (<1.0 mg/l) has gradually been introduced in the endemic villages [20]. By 2008, public water (tap water or mountain spring water) replaced well water in more than 93% of the villages. Few or no studies have evaluated the health impacts of this water change policy. It is yet to be determined whether children’s health in these endemic fluorosis areas has reached the level of children living in non-fluorosis areas. In particular, no studies have examined bone metabolism changes after the intervention of supplying low fluoride water, especially with regard to the changes in various biomarkers (exposure and bone damage). In order to fill these knowledge gaps, this study compared water fluoride, urinary fluoride (a measure of internal exposure), and four bone metabolism indicators including serum osteocalcin (BGP), calcitonin (CT), alkaline phosphatase (ALP) and bone mineral density between the four intervention villages and one control village.

Assessing endemic fluorosis is a daunting task for local health departments. The key is to determine the allowable limit of the water fluoride levels. Before the endemic fluorosis occurs, early indicators of bone damage such as BGP, CT, ALP and bone mineral density change. Therefore, it is important to determine the thresholds for these bone indicators to assess early fluoride impact to human health. In this study, the dose - response relationship model was used to estimate safe doses of water fluoride and urinary fluoride using the above bone-related indicators, as well as to identify the threshold relevant to bone metabolism indicators. Finally, by evaluating the relationships between the urinary fluoride (UF) and the four bone indicators, we identified the most sensitive and reliable bone metabolism indicators to use in intervention efforts in endemic fluorosis areas.

## Methods

### Study population and design

This study was conducted in four of 13 endemic fluorosis villages (intervention villages) in Chaonan District of Shantou, China, randomly selected for the observation group based on the years of having a public water supply. The selected villages were Xiangang village (A) of Sima town, Fanxi village (B) of Chendian town, Yiying village (C) of Xiashan town and Xibei village (D) of Chendian town. Shangnan village (E) of Jingdu town served as the control village and was selected based on its non-endemic fluorosis status as well as its similarity in demographics (annual average income per person: RMB 5,000 yuan and all rural, farming communities), dietary habits and life styles (eating porridge, rice, fish, and drinking a lot of high concentration of “Wulong” or “Tieguanyin” (Wulong green tea), similar geographic areas (all living within 10 KM from Lian river), similar health status (no acute infectious diseases and chronic diseases including bone metabolism-related diseases), and similar medical insurance (New Cooperative Medical System sponsored by local government), in villages A, B, C and D. The investigation was conducted between October 18, 2009 and November 15, 2009. A total of 600 school children (6-12 years old) were selected who had resided since birth in their village of residence. Sampling methods included:1) randomly selecting one school from each village, 2) randomly selecting one class from grades 1-6 from each selected school, and 3) randomly selecting 20 children (50% boys and 50% girls) from each selected class. All the children in the sample were measured for UF, serum ALP, BGP, CT and bone mineral density. Blood and urine samples were collected following approval by the Medical Ethics Review Group of Sun Yat-sen University School of Public Health and informed consent from the principals of the schools and the parents of the children.

### Determination of water and urinary fluoride

Drinking water samples were collected from peripheral taps around the village and the center point of each village. Fifty ml of each sample was collected in a clean and dry polyethylene plastic bottle. The concentration of drinking water fluoride was determined in the laboratory using the ion electrode test based on the China’s Drinking Water Standard Detection Method GB/T5750-2006 [21]. Urine samples were collected from participating children at 11:00 am, and 50 ml of each sample was sent to the laboratory. Urinary fluoride content, an indicator of long-term fluoride exposure, was determined using the fluoride ion electrode method based on the China’s Urinary Fluoride Detection-Fluoride Electrode Method WS/T89-1996 [22].

### Quality control for water fluoride and urinary fluoride

Quality control of laboratory equipment was ensured by regular instrument calibration [21,22]. Under the guidance of the National Drinking Water Fluoride Content Determination, the standard curve regression equation for water fluoride determination is: $\hat{y}=-311.34+52.971x\left(r^{2}=0.9993\right)$. The spiked recoveries of different potential values ranged from 96.3%-102.0% (Normal Value is between 95.0% and 105.0%). In addition, the standard curve regression equation for the determination of fluoride in urine is: $\hat{y}=25.118-348.89x\left(r^{2}=0.9991\right)$. The relative error for 10 synthetic urine samples (1.25 mg/L) is E=3.07%.

### Measurement of bone metabolism indicators

#### Serum BGP, CT and ALP testing and bone density measurement

Venous blood samples (5 ml) were obtained using a vacuum blood tube from the upper arm of each participating child. Blood and urine samples were collected simultaneously and at the same time of day for each village. It took five days to complete this sample collection (one day per village). The blood samples were placed in the laboratory refrigerator at a constant temperature of 4°C. Two milliliters of serum was separated from each blood sample next day and kept in the laboratory freezer at a constant temperature of -20°C. Serum osteocalcin (BGP) and calcitonin (CT) contents were determined using radioimmunoassay (The intelligent Montioring γ radioimmunoassay instrument (SN-695B) was from Rihuan instrument of Shanghai nuclear research, China, a kit was developed by Hengda Biological Technology Development Co., Ltd, Nanjing, China.). The alkaline phosphatase (ALP) activity was detected using benzene disodium phosphate method (a kit from Nanjing Jiancheng Biotech Company in China). Bone mineral density was measured by dual energy X-ray bone densitometer (DTX-200 US Osteometer MediTech, Inc. Signal Hill, USA) with the accuracy of one ten thousandth (g/cm^2^), The detection sites of bone mineral density were the ulna and radius of the forearm.

### Quality control and standard curves drawing

According to the concentration from low to high, six concentration groups were pooled to generate an average value and to calculate the coefficient of variation (*CV*)%. For instance, the *CV*% of BGP for the dose groups (from low to high) were 1.55%, 0.37%, 0.22%, 4.35%, 3.66% and 1.00% respectively. The *CV*% of CT for the dose groups (from low to high) were 0.49%, 0.80%, 1.17%, 1.13%, 2.77% and 1.94% respectively. The standard curve was drawn based on the average attenuation counts. Every sample for BGP, CT and ALP determination had its own parallel sample (one sample divided into two for the same measures at different times to ensure the reliability of the measurement results, as well as to prevent system errors). All the coefficients of variation were less than 10%.

### The abnormal criteria of four bone metabolism indicators

The 90th percentile among children of the same gender and age in the control village was used to identify participants as normal or abnormal. Each individual’s bone mineral density’s *Z* score was calculated using the formula: *Z* score = (*Xμ* − *Xm*)/*s*, where *Xμ* = individual bone mineral density; *Xm* = mean value of the control village (Village E) group’s bone mineral density (stratified by age and gender); and *s* = standard deviation. The abnormal *Z*-value is *Z*<-2, from which we could obtain the abnormal incidence of different doses for all the effect indicators.

### Data analysis

All data were presented as mean values with standard deviations (SD). Statistical tests for significance were performed using the one-way analysis of variance (ANOVA), Kruskal-Wallis test, *t* test, Chi-square test and Chi-square test for trend with SPSS ver. 17.0 software (SPSS, Chicago, IL). Differences were considered to be statistically significant at *P* < 0.05. While F-values were found being significantly different among all intervention villages combined compared to the control village, pair-comparison tests were conducted to further determine which intervention village was different from the control village. Benchmark dose (BMD) modeling was conducted using USEPA’s BMD Software v. 2.2 to obtain dose-response relationships between the urinary fluoride and the four bone indicators. Benchmark Response(BMR)was used as a predetermined incidence of adverse response that determines the Benchmark dose [23]. A BMR of 10% was used to obtain four bone indicators along with appropriate 95% lower confidence limits (BMDL_10_). The best fitting model was selected from the multi-models based on the Akaike information criterion (AIC).

## Results

### Comparisons of water -fluoride and urine-fluoride levels between villages

In total, 550 children provided urine samples and of these, 24 children did not provide blood samples. Thus, there were 526 children who provided both blood and urine samples in the 5 villages studied. The four intervention villages (A-D) received low-fluoride public drinking water (< 1.0 mg/L) over a period of 6-17 years. The levels of fluoride in the water prior to this period were compared to the water-fluoride levels measured following the water supply replacement (Table 1) as well as the level in the control village (Village E). The data showed that the concentration of fluoride in the control village changed minimally during this period while the fluoride levels in drinking water at the intervention villages (B, C and D) declined by 95.8%-98.0% ,to levels similar to the control village (0.11 mg/l) (*t*=1.567, *P*=0.215).

**Table 1:** Water fluoride levels before and after the change of public water supply, Guangdong, China, 1992 to 2009

	**Change of public water supply**		
**Village**	**Years of using public water**	**Before water F (mg/L)**	**After water F (mg/L)**
A	6	5.51	0.11
B	14	2.17	0.09
C	15	3.99	0.11
D	17	3.31	0.10
E	Control	0.12	0.11

Urinary fluoride levels (UF) in intervention villages were compared to those in the control village using the Kruskal-Wallis test (Table 2). In general, the pooled average UF levels (0.430±0.380 mg/l) in the four intervention villages were significantly lower or similar to the values in the control group (0.480 ± 0.290 mg/l)(*P*<0.001). Compared with the control village, children living at Village C (0.360 mg/l) and D (0.270 mg/l) showed significantly lower UF levels after receiving 15-17 years’ low fluoride water supply using rank transformation and Dunnett t-test. Children in Village B had similar UF levels as those in the Control village after 14 years of water change (no statistically significant differences). However, the average UF levels for village A (0.620 ± 0.380 mg/l) (after 6 years of intervention) were still significantly higher than the control village E. Another interesting finding is that urinary fluoride levels are higher than drinking water fluoride levels (Table 1 & Table 2).

**Table 2:** Urinary fluoride levels after change of water supply, Guangdong, China, 1992 to 2009

**Village**	**Years of using public water**	**# of samples**	UF(mg/l) ^*^
A	6	128	0.62 ± 0.38^#^
B	14	108	0.51 ± 0.42
C	15	73	0.36 ± 0.12^#^
D	17	114	0.27 ± 0.28^#^
E	Control	127	0.48 ± 0.29
Total	-	529	0.44±0.35
Median^*^			0.43±0.38

*: In Shapiro-wilk test, urinary fluoride levels were not normally distributed (*P*<0.05). Kruskal-Wallis test shows five Villages were statistically significant: χ^2^=127.92, *P*=0.00.

^#^: Dunnett t-test method after rank transform for the pairwise comparisons, the difference was statistically significant (*P*<0.05).

*: Pooled Median from group A through D; *P* values of Kolmogorov-Smirnov Z test for median difference between pooled median and control median; *Z*=1.63, *P* =0.01.

### Comparison of BGP, CT, ALP and bone mineral density between villages

The average serum levels of biomarkers Osteocalcin (BGP), Calcitonin (CT) and Alkaline Phosphatase (ALP) among the children in each of intervention villages A, B, C and D were compared to control village E (Table 3). The pooled mean serum level for the four intervention villages was also used for comparison to the control village. While the pooled mean from Group A through D, biomarkers BGP and ALP were significantly lower when compared to the control village E (*P*<= 0.01), but the mean of CT and Bone Density among the 4 intervention villages were similar to those in the control village.

**Table 3:** Comparison of BGP、 CT、 ALP and Bone Mineral Density between the Intervention Villages and the Control Village after Chang of Water Supply, Guangdong, China

**Village**	BGP(μg/L)^*^		CT(pg/ml)^*^		ALP(U/L)^*^		**Bone density (g/cm**^**2**^)^*^	
	***n***	$\bar{\chi }\pm s$	***n***	$\bar{\chi }\pm s$	***n***	$\bar{\chi }\pm s$	***n***	$\bar{\chi }\pm s$
A	119	16.19±3.18	120	256.44±103.39^#^	117	29.88±9.02	98	0.29±0.06
B	98	13.81±2.40^#^	98	156.81±57.82^#^	98	33.06±15.79	77	0.26±0.04
C	73	15.77±3.45	63	126.44±39.49^#^	73	27.02±7.56^#^	71	0.28±0.07
D	109	14.23±2.56^#^	111	179.48±57.66	108	25.39±7.06^#^	95	0.29±0.07
E	127	15.81±2.77	127	197.21±51.29	127	31.90±10.15	113	0.27±0.04
*F*		14.465		48.959		9.831		3.781
*P*		0.000		0.000		0.000		0.005
Normal range^%^		(10.38,21.24)		(96.68,297.74)		(12.01,51.79)		(0.19,0.35)
Mean^*^		15.14±2.90		190.80±72.84		29.21±10.53		0.28±0.06
P^**^		= 0.01		> 0.1		< 0.01		≈ 0.1

*: By Shapiro-wilk test, BGP ,CT ,ALP and Bone Density are normal distribution(*P*>0.05).

^#^: Pairwise comparisons using Dunnet *t* test method with the control village E, the difference was statistically significant (*P*<0.05).

^%^:Normal ranges were calculated according to the control village.

*: Pooled mean from group A through D.

^**^: *P* values of *t* test for mean difference between pooled mean and control mean.

Significant differences were seen between the intervention and control villages for all biomarkers and indicators in the F tests (all *P < 0.05*, Table 3). Except for Village A and C, the children’s serum BGP levels in Villages B and D were significantly lower than that in the village E in pairwise comparison (*P < 0.05*). For CT levels, village A was higher than E, while village B and C were lower than E. The ALP levels in the villages C and D were significantly lower than E. Total bone mineral density in pairwise comparisons showed no difference between the intervention and control villages.

### Analysis of urinary fluoride and bone metabolism indicators

The relationships between increased UF concentrations and abnormal bone indicators are described in Table 4. The abnormal thresholds of the three bone indicators including BGP (>19.24 μg/l), CT (>254.15 pg/ml), and ALP (>46 U/l) were defined according to the 90% upper limit of the same indicator with similar gender and age of children in the control village. Bone mineral density was defined as abnormal if the bone density *Z* value was<-2. All bone indicators were analyzed by dividing the sampling children into 4 groups according to their urinary fluoride contents (mg/l) as follows: 0 -<0.3, 0.3-<0.6, 0.6- <0.9 and 0.9- (Table 4). From Table 4 we could find that the highest percentage of the participating children with abnormal bone indicator is CT (13.7%), followed by BGP (10.1%) and then ALP (8.8%), and the lowest rate was bone mineral density (3.3%). The linear trend test using χ^2^ test showed that with the increase of urinary fluoride, the abnormal rates of BGP and CT also increased, but no such linear trends for ALP and bone mineral density with UF were observed.

**Table 4:** Urinary Fluoride and Abnormal Incidences of Bone Metabolism Indicators among the UF concentration groups

**UF(mg/l)**	**BGP**		**CT**		**ALP**		**BMD**	
	**n**	**Abnormal(%)**	**n**	**Abnormal(%)**	**n**	**Abnormal(%)**	**n**	**Abnormal(%)**
0.0 ~	120	9(7.5)	119	10 (8.4)	119	11(9.2)	98	2(2.0)
0.3 ~	252	22(8.7)	245	32(13.1)	245	17(6.8)	221	8(3.6)
0.6 ~	96	14(14.6)	96	17(17.7)	96	13(13.5)	81	1(1.2)
0.9 +	58	8(13.8)	59	12(20.3)	59	5(8.8)	54	4(7.4)
Total	526	53(10.1)	519	71(13.7)	519	46(8.8)	454	15(3.3)
χ^2^		0.42		6.42		3.96		1.46
*P*		0.22		0.09		0.27		0.70
Trend test χ^2^		3.52		6.85		0.45		1.60
*P*		0.03		0.00		0.25		0.10

### Dose - response relationship between urinary fluoride and bone metabolism indicators

**Figure 1:**
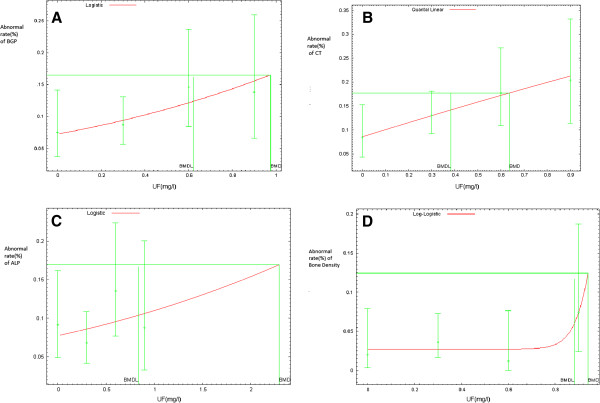
A-D The Dose-response Relationship between Urine Fluoride and Bone Metabolism Indicators, Guangdong, China from 1992 to 2009.

We selected different analysis models to assess the dose-response relationship between UF and four different bone indicators based on data distribution and model fitness (Figure 1). The Logistic regression model used to describe the dose - response relationship between urinary fluoride and BGP was: *logit(P)=-2.553+0.955UF*. The Quantal-Linear model was selected to describe the dose - response relationship between urinary fluoride content and CT, which was *P=0.085+0.915(1-exp(-0.166UF)).* A logistic model of dose-response relationship for urinary fluoride and ALP was described as: *logit(P)=-2.48+0.388UF*, and the model for urinary fluoride and bone mineral density was Log-Logistic; the model used is: *P=0.03+0.97(1+exp(1.09-18ln(UF)))*^*-1 *^(Figure 1:A-D). All the regression models passed the goodness fit test and the results showed that they fit well (*P>0.05*).

Among these four indicators, the Benchmark dose of CT is the most sensitive one. When the abnormal rate of it increases to 10%, the corresponding benchmark dose is 0.636 mg/l and 95% lower limit is 0.383 mg/l, which means when the urinary fluoride changes to 0.383 mg/l, the CT may undergo noteworthy abnormal changes. The second most sensitive indicator is BGP (BMD and BMDL were 0.975 mg/L and 0.622 mg/l respectively), i.e., when the urinary fluoride changes to 0.622 mg/l, the risk of BGP anomalies will reach 10%. The least sensitive indicators are ALP (BMDL: 0.837 mg/l ) and bone mineral density (BMDL: 0.881 mg/l) (Table 5). The dose - response curve of bone mineral density (Figure 1) is not linear and quite different from other indicators, which shows that at lower doses of urinary fluoride, the anomalies of bone mineral density changed little, but when the urinary fluoride increased to a certain extent, the anomalies of bone mineral density changed significantly.

**Table 5:** The BMD and BMDL of urine fluoride between the abnormal incidences of bone metabolism indicators

**Village**	**Indicator**	**Model**	**χ2**	***P***	**BMD (mg/L)**	**BMDL (mg/L)**
A	BGP	Logistic	0.82	0.66	0.98	0.62
B	CT	Quantal-linear	0.05	0.98	0.64	0.38
C	ALP	Logistic	3.34	0.19	2.30	0.84
D	Bone density	Log-logistic	1.51	0.47	0.94	0.88

**Figure 2:**
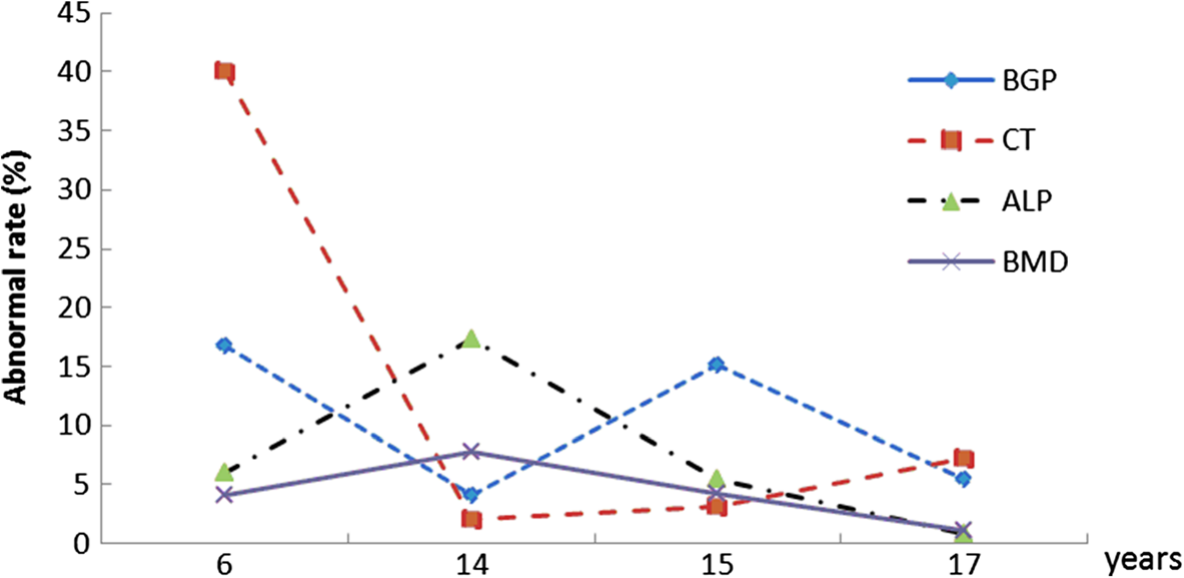
Time Trends of Abnormal Rates in Bone Metabolism Indicators after the Change of Public Water Supply, Guangdong, China from 1992 to 2009.

This study showed that with six or more years of changed water fluoride levels, all the abnormal bone indicators except for CT had declined to low levels (Figure 2). However, abnormal CT did not decline till around 10-14 years after the intervention. Bone mineral density showed the lowest in all bone indicators and was relatively stable over time.

## Discussion

This study found that the water fluoride levels in villages (A-D) after the water source change were much lower than the average pre-intervention level (3.475 mg/l) and the post-intervention levels in all intervention villages declined to 0.11 mg/l or lower. These reduced fluoride levels are lower than the Chinese National Fluoride Standard (1.0 mg/l), and the World Health Organization (WHO) recommended drinking water fluoride levels of 0.5~1.5 mg/l. The fluoride concentration of drinking water is the only objective indicator reflecting drinking water source of endemic fluorosis and environmental fluoride, and for evaluating the policy effect of water change/treatment in the endemic fluorosis areas. As most fluoride intake in the endemic fluorosis areas of China were from drinking water, it is appropriate to monitor the fluoride level in drinking water in endemic areas and over time.

We also found that urinary fluoride levels among the residents living in the villages after 14-15 years of water source change were similar or lower than the level in the control village. In other words, over the years that the high fluoride water was replaced by lower fluoride water (public water), urinary fluoride excretion had gradually decreased. Urinary fluoride (UF), is an objective measure of fluoride intake, because it is related to the water fluoride and reflects the human level of fluoride metabolism [24]. Our study also found that the urinary fluoride concentrations declined the longer the villages had access to the public water supply, which was supported by similar results found in other studies [25,26]. Gongju Yin et. al. conducted a survey among children aged 8-12 in the fluorosis areas and found that drinking water de-fluoridation resulted in water fluoride concentration reduction and consequent reduction in urinary fluoride [27]. The results from these prior studies are consistent with the findings of our study. The level of urinary fluoride among children in intervention villages having a public water supply for more than 10 years (villages B, C and D) was similar to the level of UF in the control village. However, the children’s UF in village A is still higher than the control village probably due to a shorter time (6 years) of having a public water supply. An interesting finding from our study was that urinary fluoride levels are higher than drinking water fluoride which may be due to the accumulation of fluoride in the body; i.e., fluoride released from bone or other body tissues and excreted through the kidneys. It could also be due to the body accumulation of fluoride from other sources such as tea, local-grown vegetables, fruits or animals. This process could take place over a long period of time.

Long-term intake of excessive fluoride has adverse effects on health including dental fluorosis, change of bone metabolism indicators, and finally bone damage [28-32]. After a long period of low-fluoride public water exposure, all the indicators had reached to the normal level after the water changed for six years or more except for CT. These findings imply the success of the policy of fluoride water change and are consistent with the relationship between urinary fluoride and fluoride-induced bone metabolism indicators including BGP, CT, ALP, and bone mineral density reported by one of the few studies in this area [33,34].

To select the best bone metabolism indicators related to high fluoride exposure, this study also examined the dose-response relationship between children’s urinary fluoride level and abnormal bone indicators. We found that children’s abnormal levels of BGP and CT increased as the levels of their urinary fluoride contents increased, but this dose-response relationship was not as apparent with respect to serum ALP levels and bone mass density. All of these four indicators can reflect the impact of fluoride on bone metabolism, including bone formation and bone resorption, which is accelerated and is the reason of osteosclerosis and bone softening due to the skeletal fluorosis.

Serum BGP, CT and ALP are commonly used as markers reflecting bone generation. As Liang et.al. described [35], BGP, a collagen protein produced and secreted by osteoblasts, could regulate the bone metabolism through binding with hydroxyapatite (HA) to form fluoride phosphate stone. This results in the increase of BGP secretion, destruction of normal bone mineralization rate, and induction of osteomalacia and osteoporosis [36]. Therefore, serum BGP is a sensitive, specific, accurate and simple biochemical indicator to measure instantaneous change of bone metabolism [37-40]. ALP has been considered as a marker of osteoblast activity. Fluoride can affect ALP activity in two ways. i.e., changing the structure of the enzyme to directly affect the activity of the enzyme, and accumulating fluorine in the bone to promote bone cells in mitosis, and thus influence vitality of the bone cells. Therefore, the increase of serum ALP, to some extent, reflects an active cell proliferation of the osteoblasts, and suggests that the body that may have fluorosis-related damage. Wan stated that after exclusion of other metabolic bone diseases, elevated serum ALP can be used as a diagnostic indicator of skeletal fluorosis [41]. Liu has shown that when the fluoride concentration in drinking water reaches 0.58mg/l-1.59 mg/l, ALP activity is increased [42]. When the water fluoride concentration is between 1.60mg/l and-3.37 mg/l, ALP activity decreases slowly. It could decrease to as low as 57% of the normal amount, but it can return to normal levels some time later [43]. ALP has been thus recognized as an early and important indicator in the evaluation of bone formation and bone turnover. In addition, the main role of CT is to stimulate osteoblast formation and indirectly affect bone metabolism [44,45]. The sensitivity and specificity of CT are lower than those of BGP and ALP. Finally, bone mineral density, reflecting per unit volume of bone mineral content, is an important quantitative indicator for evaluating bone mass. Skeletal fluorosis caused by osteoporosis reduces per unit volume of bone mass, i.e., decreases the bone mineral density. The measurement of bone mineral density can be used as a more objective and accurate evaluation indicator of bone changes [46]. In summary, BGP, ALP, and CT reflect pre-bone formation status, but bone mass density indicates final bone mineral change. Thus, use of all these four indicators not only demonstrate early changes of bone metabolism, but also describe the final mineral characteristics in order for us to evaluate the new regulation of supplying public water in the endemic fluorosis areas.

The benchmark dose–response models were first mentioned by Crump [23]. A BMD is defined as the dose that corresponds to a specified change in adverse response compared to the response in untreated situation. The dose is associated with a given incidence (e.g., 1%, 5% or 10% incidence) of effect, the Benchmark Response, based on the best fitting dose-response curve in the region of the dose-response relationship where biologically observable data are available. The resulting BMD in this study is termed BMD10 for a 10% incidence.

Because these four bone metabolism indicators are the physiological continuity indicators of the bone metabolism or damage, we need to identify a cut-off point or threshold to define abnormal value based on the dose-response relationship. This study found that CT has the lowest threshold or BMD among all bone metabolism indicators, but ALP has the highest BMD. Unfortunately, no available literature can be found to compare with our findings. This study also found that most bone damage markers went back to normal level after six years of water change intervention except for CT which significantly declined after 10-14 years. Bone mineral density is the most stable indicator. No other studies are available to compare with our findings.

This study is one of the only or few research efforts to evaluate how this environmental intervention affected public health. We not only examined fluoride change (external exposure marker) in public water after providing low fluoride water, but also assessed UF (internal exposure marker) and bone damage related to fluoride exposure (health markers). Different from some prior studies which relied completely on self-reported information, use of these comprehensive biomarkers from exposure to health endpoints minimized reporting bias and provided reliable data for intervention evaluation. This study may also be the first to describe and compare the changes of several pre-clinical and clinical bone metabolism markers after intervention, which could help in understanding the potential biological mechanism and identify sensitive or appropriate health indicators for future public health surveillance.

One of the major limitations in this study is lack of baseline data for UF and bone metabolism indicators prior to water source change. However, a control village was carefully selected for comparison based on similar status as the intervention villages (A-D) on demographic characteristics (annual income, insurance type, and all rural, farming communities), dietary habits (eating porridge, rice, fish, and drinking a lot of high concentration of tea), similar geographic areas (all near river), and similar health status for participant students by excluding those with infectious diseases and bone metabolism disorders. In other words, all living conditions between the intervention villages and the control village were similar except for water sources. In this study, since the public water supply to all fluorosis endemic areas had similar concentrations of fluoride, and the fluoride levels in all intervention villages were reduced to levels similar to the non-fluorosis endemic areas, it is not possible to assess the dose - response relationship between water fluoride and the indicators. Nevertheless, we did use “the years of having the new water source” as an indicator to assess the health impact of duration of water intervention. In addition, as most previous studies in this area were conducted in China or other countries, limited English literature was available to compare with our findings. Finally, although this study demonstrated the success of the environmental intervention program, i.e., providing low-fluoride water supply in all endemic villages in Guangdong province, similar or even lower values of fluoride in some intervention villages compared to the control village or under the national health standards (1.0 mg/l) caught the attention of local environmental and public health agencies with respect to dental caries. Therefore, the possibility that the drinking water with very low concentration of fluoride could induce dental caries in these area and whether fluoride levels in the water supply of these villages should be modified, is worthy of the further study.

## Conclusion

In summary, when public water with low fluoride concentration was supplied to endemic fluorosis villages for more than 6 years, the children’s urinary fluoride decreased to the normal range. Urinary fluoride content is reduced more the longer the duration of the change in the public water supply. Consistently, almost all the fluoride-induced bone metabolism indicators of the study population in the intervention villages have reached the normal level or levels lower than those of the control village. Among all the bone metabolism indicators, BGP and CT are the most sensitive indicators related to UF based on BMD, but abnormal bone mineral density was the lowest overtime and showed the most stable change after the intervention.

## Abbreviations

ALP: Alkaline phosphatase;BGP: Osteocalcin;BMR: Benchmark response;BMD: Benchmark dose;BMDL: Benchmark dose lower-limit;CT: Calcitonin;UF: Urinary fluoride

## Competing interests

No conflict of interest has been declared by the authors. None of the authors’ spouses, partners, or children have financial relationships that may be relevant to the submitted work.

## Authors’ contributions

SC and BL were responsible for the study conception and design. YH,XZ,JW and XF performed the data collection. MZ provided statistical expertise. YX was responsible for experimental analysis. SY supervised the study. SC was responsible for the drafting of the manuscript. Dr. SL and SH provided critical comments on methodology issues and made significant revisions to the paper for improving its quality. SY, the corresponding author had final responsibility for the decision to submit for publication. All authors read and approved the final manuscript.

## Pre-publication history

The pre-publication history for this paper can be accessed here:

http://www.biomedcentral.com/1471-2458/13/156/prepub
